# Reverse development of vaccines against antimicrobial-resistant pathogens

**DOI:** 10.1038/s41541-024-00858-4

**Published:** 2024-04-03

**Authors:** Fabio Bagnoli, Ilaria Galgani, V. Kumaran Vadivelu, Sanjay Phogat

**Affiliations:** 1 GSK, Siena, Italy; 2 GSK, Rockville, MD USA

**Keywords:** Clinical trial design, Biologics

## Abstract

Vaccine R&D is typically a lengthy process taking >10 years. However, vaccines still fail in clinical development because of unreliable animal models or absent immunological correlates of protection. Without a correlate of protection, phase-1 and -2 studies of safety and immunogenicity can fail to predict phase-3 efficacy. Indeed, the history of vaccine development is replete with promising phase-1 and -2 results and failed phase-3 efficacy trials. To avoid this misfortune, we present *Reverse Vaccine Development* for vaccines against antimicrobial-resistant (AMR) pathogens. In this approach, instead of evaluating efficacy in phase 3, proof-of-principle efficacy is evaluated as early as possible in a population with a high incidence of disease, which may differ from the population intended for registration, and can be a controlled human infection population. To identify a correlate of protection in these populations, the vaccine-elicited immune response is compared between protected and unprotected subjects. If a correlate is identified, it can help to refine the vaccine dosage, schedule, and formulation, and facilitate the assessment of vaccine efficacy in other populations with different attack rates, subject characteristics, and disease manifestations. This may be the only way to provide life-saving vaccines to populations affected by AMR-pathogen diseases at incidences that are typically low and unsuited to phase-3 efficacy trials. The availability of a correlate of protection early in clinical development can potentially prevent failures of large phase-3 trials and unnecessary exposures of populations to inefficacious vaccines that have resulted in disinvestment in the development of vaccines against AMR pathogens.

## Introduction

A recent estimation of the impact of antimicrobial resistance (AMR) on global human health has indicated a medical need comparable and likely larger than HIV and malaria. Unfortunately, developing vaccines against AMR pathogens is being hindered by a lack of understanding of their correlates of protection. Reverse Vaccine Development, which we are pioneering along with the development of a vaccine against *Staphylococcus aureus*, is proposed as an approach for easing and accelerating the clinical development of vaccines against AMR pathogens.

Vaccine development can be long, difficult, and costly. However, it becomes easier when a correlate of protection is known. For example, the speed of SARS-CoV-2 vaccine development was not only due to the extraordinary effort of companies and health authorities, but also to knowledge of the likely mechanism of protection: antibody to the spike protein preventing interaction with the host cell. Hence, neutralizing antibody titers became the common thread linking preclinical and clinical development that guided the selection of vaccine candidates eliciting high neutralizing antibody titers in mice; and subsequently, high neutralizing titers were shown to correlate with clinical efficacy. Recently, SARS-CoV-2 vaccines have been found to induce memory CD8^+^ T-cell responses in the first week of breakthrough infection that correlate with control of infection^1^.

Unfortunately, for many human pathogens, including those on the World Health Organization’s (WHO) list of global priority pathogens of antibiotic-resistant bacteria, the mechanism of protection remains unknown, and candidate vaccines against these pathogens have failed (e.g., *S. aureus* and *Pseudomonas aeruginosa*). Nonetheless, vaccines against AMR pathogens continue to be developed, supported by genomics, proteomics, and immunomics to identify antigens. However, without a correlate of protection, late-stage clinical development is risky: many thousands of research participants may be needed for efficacy evaluations, and after many years of R&D and large expense, the trials may fail.

During the development of a vaccine candidate against *S. aureus*, we realized the need for a new vaccine development paradigm wherein data generation on efficacy and the immune response, for identifying a correlate of protection, should occur early, instead of in phase 3, reversing the typical order and why we named the approach Reverse Vaccine Development. This approach relies on the availability of a population with a high rate of disease, which may differ from the population intended for registration.

The demonstration of efficacy (proof of concept) in the high attack-rate population enables a comparison of the protected and unprotected subjects for their immune response to the vaccine—to identify a correlate of protection. This approach is especially important when animal models are unreliable for determining correlates of protection. Knowledge of the correlate of protection in early clinical trial phases may prevent failures of large phase-3 trials, unnecessary exposures of populations to inefficacious vaccines, and disinvestment in the development of vaccines against AMR pathogens. Reverse Vaccine Development has the potential to transform the R&D of vaccine candidates against AMR pathogens that lack an animal-model-derived correlate of protection.

## Standard clinical trial design

Standard clinical study designs for vaccines start with a phase-1 assessment of safety and immunogenicity in ~50-300 healthy volunteers. In phase 2, these same endpoints are evaluated in a few hundred subjects in the target population intended for registration, and early evaluation of efficacy endpoints may also be included. Finally, phase-3 trials assess efficacy and further assess safety and the immune response in the targeted population, typically in thousands to tens of thousands of subjects.

Phase 3 trials can be very large, and without a correlate of protection they are at higher risk for failure. For example, when the *Streptococcus pneumoniae* heptavalent vaccine was being developed, the surrogate of protection, opsonophagocytosis, had not yet been established. Additionally, the incidence of streptococcal pneumonia and invasive pneumococcal disease was relatively low in the target population. Therefore, a very large phase-3 efficacy study of >84,000 subjects was needed. Large phase-3 trials for preventive vaccines are unfortunately a common problem (Table 1).

**Table 1:** Sample sizes of exemplary pivotal efficacy trials for vaccines against various infectious diseases

Vaccine	Target pathogen	Target population	Primary clinical endpoint	Sample size	Number of centers	Study outcome	References
PCV13 (13 valent pneumococcal polysaccharide conjugate vaccine)	*S. pneumoniae*	>65 years of age	Prevention of vaccine-type pneumococcal community-acquired pneumonia (CAP), including non-bacteremic/non-invasive CAP, and vaccine-type invasive pneumococcal disease (IPD)	84,496	59 sentinel hospitals were used for the surveillance of CAP and IPD	The study achieved its primary and secondary objectives.	20 NCT00744263
SA4Ag (4 antigens: 2 polysaccharide conjugates and 2 recombinant proteins without adjuvant)	*S. aureus*	18 through 85 years of age undergoing elective open posterior multilevel instrumented spinal fusion surgery	Subjects with confirmed postoperative *S. aureus* bloodstream infections and/or deep incisional or organ/space surgical-site infections occurring within 90 days after the index surgical procedure contributed to the primary efficacy endpoint analysis.	Planned >6000; Study terminated after interim analysis with 3450 subjects enrolled	302	Study discontinued after interim analysis due to futility	21 NCT02388165
RV144 HIV vaccine: priming with Live recombinant ALVAC-HIV (vCP1521) and boosting with AIDSVAX B/E glycoprotein (gp)120 (MN and A244)	HIV (human immunodeficiency virus)	18–30 years	Prevention of HIV infection, assessed every 6 months for 3 years in HIV-uninfected adults	16,402	8	60% efficacy at 12-months 31.2% efficacy at 42 months	22 NCT00223080
IC43—recombinant *P. aeruginosa* fusion protein	*P. aeruginosa*	18–80 years	Intensive care unit (ICU) patients with a need for mechanical ventilation for >48 h	803	50	The vaccine provided no clinical benefit over placebo in terms of overall mortality	23 NCT01563263

## Reverse Vaccine Development

**Fig. 1:**
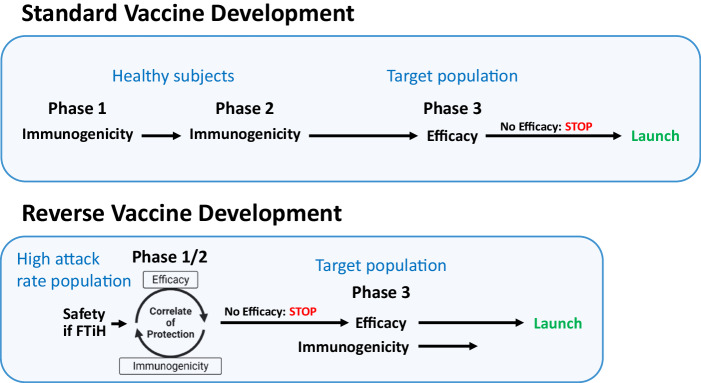
Standard vs. Reverse Vaccine Development clinical trial designs.

Reverse Vaccine Development starts with a phase 1/2 study that assesses safety, immunogenicity, and efficacy. If the vaccine is First-Time-in-Human (FTiH), a phase-1 safety lead-in study may be needed that escalates dose, without adjuvant and then with adjuvant. If no safety issues emerge, the phase-2 study can proceed to evaluate immunogenicity and efficacy (Fig. 1).

Importantly, the population used for the efficacy assessment needs to have a high attack rate. But the attack rate may not be high in the population intended for registration. Therefore, Reverse Vaccine Development requires a population with a high attack rate that is available for study.

For our *S. aureus* vaccine candidate, such a population is available: patients with a community acquired skin and soft tissue infection (CA-SSTI) whose risk for recurrence is high. Such populations are also available for other infectious diseases.

An efficacy evaluation of a candidate vaccine early in clinical development is of key importance. If the vaccine is found not efficacious, unnecessary exposure of subjects to the vaccine is avoided. If the vaccine is efficacious, correlates of protection can be explored.

How correlates of protection are studied depends on the study outcome. If the vaccine was partially efficacious, the immune response to the vaccine can be analyzed for differences between vaccinees who became infected and those who were protected to identify an immunological signature of the protective vaccine response.

If an immune correlate of protection is identified, it can help to refine the dosage, schedule, and formulation of the vaccine. A correlate of protection can also provide a benchmark for the potential of other populations to be protected by the vaccine, including the phase-3 study population. This can be of critical importance and the only way to provide lifesaving vaccines to populations where the rates of devastating infectious diseases are usually low and not suited to phase-3 efficacy trials.

## Standard clinical trial designs for *S. aureus* vaccines failed to demonstrate efficacy

*S. aureus* is a leading cause of community acquired skin infections, surgical site infections, bacteremia, and pneumonia. These infections are associated with a massive burden of morbidity, mortality, hospital length of stay, and patient cost. Additionally, *S. aureus* rapidly acquires antibiotic resistance, which seriously threatens human health. Current antibiotics and care bundles leave a tremendous unmet medical need worldwide. Further, there are no licensed vaccines on the market, despite the significant efforts of public and private initiatives.

Four vaccine candidates against *S. aureus* have been evaluated in clinical efficacy trials: (1) StaphVAX, a conjugate vaccine developed by Nabi Biopharmaceuticals, targeting capsular polysaccharides type 5 (CP5) and 8 (CP8)^2^; (2) V710, a vaccine targeting the iron-scavenging protein IsdB, developed by Merck^3^; (3) the Pfizer-developed, four-component vaccine candidate, SA4ag (containing CP5, CP8, ClfA, and MntC)^4^; and (4) NDV-3A, a vaccine targeting the Als-3 adhesin/invasion protein from *Candida albicans* that has structural similarity to the *S. aureus* protein clumping factor A (developed by Novadigm)^5^. These candidates were advanced to clinical development based on in-vitro assays and mouse models, despite their unknown translatability to humans. Their clinical development used a standard design, assessing safety and immunogenicity in healthy subjects in phase 1 and 2, followed by the assessment of efficacy in the target population in phase-3 trials. The phase-1 and -2 trials raised no safety concerns, and the elicited antibody was significantly greater than control and included functional antibodies. Despite this promise, the phase-3 efficacy trials failed. The high economic losses and the exposure of many subjects to the inefficacious vaccines resulted in disappointment and disinvestment.

## Reverse Vaccine Development for evaluating our *S. aureus vaccine* candidate

**Fig. 2:**
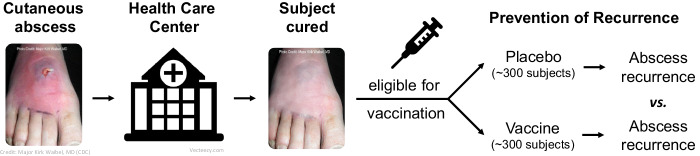
Paradigm clinical trial design (phase 1/2) in Reverse Vaccine Development: S. aureus vaccine in subjects at high risk of SSTI.

To avoid an extensive clinical trial that evaluates immunogenicity that may not predict efficacy, we are engaged in the early evaluation of the efficacy of our vaccine candidate in individuals who were recently cured of *S. aureus* community acquired skin and soft tissue infection (CA-SSTI) and are at high risk for recurrence — using prevention of recurrence as the efficacy endpoint (Fig. 2). Because this population’s attack rate is high, the efficacy evaluation requires relatively few subjects. Additionally, CA-SSTI rarely has a severe outcome. Further, CA-SSTI is the most common disease associated with *S. aureus* in communities and hospitals. These characteristics make CA-SSTI patients a good population for a proof-of-principle efficacy evaluation.

First, to characterize the CA-SSTI population, we confirmed the high CA-SSTI recurrence rate by a retrospective chart review of cases in patients aged ≥18 years at three US medical centers between 2006 and 2016^6^. We identified index cases (i.e., first occurrence cases) in one calendar year that we assessed for any recurrent infection in the following 12 months. Most cases were without key comorbidities. Across the centers, 16.4%–19.0% of the index cases recurred one or more times.

Based on these data, we designed a clinical trial defined as phase 1/2, observer-blinded, randomized, and placebo-controlled—to assess the safety, immunogenicity, and efficacy of GSK’s *S. aureus* vaccine candidate (NCT04420221, clinicaltrials.gov). This paradigm study in Reverse Vaccine Development comprises two epochs, (1) a lead-in safety study in which the vaccine is administered to healthy adults, and (2) an immunogenicity and efficacy study in adults with a recent history of CA-SSTI. The safety lead-in study uses staggered enrollment of four groups, eight healthy subjects per group, consecutively enrolled if no safety issues are identified in the prior group. In the second epoch, the index cases of CA-SSTI are enrolled. The first 40 CA-SSTI subjects are evaluated for safety. The absence of safety issues in this group triggers enrollment to accrue a total of ~300 subjects who receive the vaccine and ~300 who receive placebo, for assessing efficacy of the vaccine in reducing CA-SSTI recurrences vs. placebo (with 0% as the Lower Limit of the 85% Confidence Interval). In addition, subjects are assessed for total IgG titers, functional titers, and T-cell responses—for exploring a correlate of protection.

## Exploring correlates of protection: systems serology, data science/machine learning

The success of Reverse Vaccine Development depends on the number and validity of the immunological readouts. Antibody titers alone are likely insufficient. Although antibody functionality integrates several immune properties, it may poorly represent comprehensive vaccine immunity. Multiple immunological parameters are needed.

*Systems serology* offers unbiased and comprehensive data for identifying previously unappreciated processes and mechanisms. For example, immunogenicity and efficacy data from a phase-3 trial of the HIV vaccine RV144 suggest that non-neutralizing Fc functional antibodies can play a protective role against the virus^7^. For conducting such an analysis, multiple serological parameters are required (i.e., antibody titers, affinity, isotype, and functionality).

*Cellular responses* should be evaluated to understand the flavor of the induced T-cell response or if the vaccine increases the frequency of T cells (CD4 and CD8) specific for the vaccine antigens. For example, a Th-1 skewed response is assumed to contribute to the activation of phagocytes (e.g., through Inf-γ) and promote pathogen clearance, and Th-17 responses can be important for facilitating phagocytosis^8^.

*Transcriptional profiling* provides a complementary and broad view of the immune response to a vaccine. In addition to profiling the T-cell response, transcriptomics can identify signatures of innate immunity, such as those emerging in response to some adjuvants^9,10^.

**Fig. 3:**
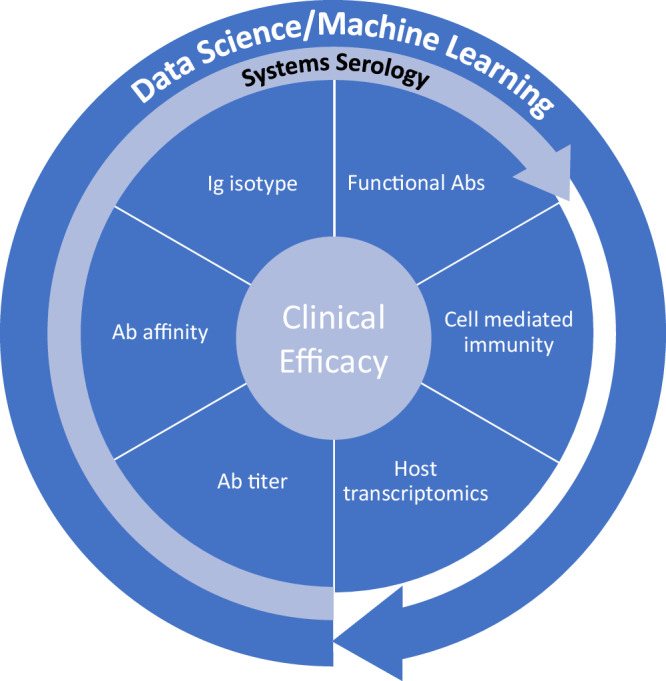
Extracting and interpreting complex data from Reverse Vaccine Development studies.

*The assessment of multiple immunological signals that correlate with each other* in mediating protection can increase the chance of identifying a signature of protection. Such patterns and associations between different readouts can be identified using machine learning, decreasing the possibility that the signature of protection is due to random chance, which is particularly important when the number of readouts is large relative to the number of protected and unprotected subjects (Fig. 3).

*Memory responses should be assessed*. While high neutralizing antibody titers or other measures of humoral immunity may correlate with protection at early times post vaccination, these signatures may wane and not inform longer term protection against disease and severe disease. Therefore, it is important to assess memory responses to breakthrough infections. For example, in the case of COVID-19 mRNA vaccination, the predominant systemic adaptive immune effectors available to limit viral replication during the first week of SARS-CoV-2 breakthrough infection were pre-existing antibodies and rapidly activated memory T cells^11^. Furthermore, in a separate study, activation of spike-specific T cells during the first week of breakthrough infection correlated with lower peak viral load and faster viral clearance^1^.

*Assessment of background immunity is crucial* as the efficacy of a vaccine and its correlate of protection may differ between an endemic population with high background immunity vs. a non-endemic population.

## Reverse Vaccine Development for pathogens lacking a correlate of protection

Reverse Vaccine Development is most suited to the development of vaccines against pathogens for which a correlate of protection is not known, such as those on the World Health Organization’s (WHO) list of global priority pathogens of antibiotic-resistant bacteria. An absence of a correlate of protection has hindered preclinical and/or clinical development of most of the CDC and WHO bacterial priority pathogens (e.g., *S. aureus*, *P. aeruginosa, Clostridium difficile*, *Escherichia coli*, *Neisseria gonorrhoeae*, *Streptococcus pyogenes*, *Klebsiella pneumoniae, Acinetobacter baumannii, Enterococcus spp., Helicobacter pylori, Candida spp., and Campylobacter jejuni*). For all these pathogens, the absence of a correlate of protection limits the meaningfulness of preclinical studies and risks clinical failure. Examples of vaccines that lacked a correlate of protection and have failed to show efficacy in clinical trials are shown in Table 2.

**Table 2:** Vaccines lacking a known correlate of protection that failed efficacy trials

Target pathogen	Main affected populations	Main diseases	Correlate of protection	Vaccines failed in efficacy trials	Reference
*P. aeruginosa*	ICU, Surgical patients (elderly)	VAP, SSI	Not known	Vaccine failed in ICU setting	23
*S. aureus*	ICU, Surgical patients, patients with central lines, patients at risk of CA-SSTI (children and elderly)	CA-SSTI, SSI, VAP, BSI	Not established yet, potentially associated with functional Ab and T-cell response	4 vaccines failed to show efficacy against BSI and SSI	2–5
*C. difficile*	Subjects at risk of CDI	Diarrhea, colitis, pseudomembranous colitis, toxic megacolon, sepsis	Not established yet, potentially associated with anti-toxin Ab	A vaccine failed to show efficacy against colitis	NCT03090191
*S. pyogenes*	Children, the elderly, and patients with underlying medical conditions	Pharyngitis, scarlet fever, blood stream infection, rheumatic heart disease (RHD), pneumonia, necrotizing fasciitis, and Streptococcal Toxic Shock Syndrome (StrepTSS)	Not established yet, potentially associated with functional Ab	In the 1940’s a whole killed bacteria-based vaccine failed to prevent respiratory infections	24

*ICU* Intensive Care Unit, *VAP* Ventilator Associated Pneumonia, *SSI* Surgical Site Infection, *CA* Community Acquired, *SSTI* Skin and Soft Tissue Infection, *BSI* Bloodstream Infection, *UTI* Urinary Tract Infection, *STD* Sexually Transmitted Disease, *CDI C. difficile* Infection.

## Requirements for using Reverse Vaccine Development

The defining concept of Reverse Vaccine Development is the evaluation of efficacy early in clinical development with the goal of identifying a proof-of-concept correlate of protection. The exploration of the correlate must be based on efficacy and immunogenicity endpoints selected to have robust readouts. Further, when the strain variability of the pathogen is significant and the vaccine targets only some strains (e.g., targeting variable polysaccharide capsule or non-conserved protein antigens), microbiology endpoints are needed. Finally, for the study to be feasible, a relatively small, high attack-rate population must be available for study, which need not be the population intended for licensure. Importantly, a correlate of protection identified in this population (training set) needs to be confirmed in a holdout set or second matched population (test set).

While the study population can be a natural high attack-rate population, e.g., the patients suffering from *S. aureus* SSTI discussed above, a controlled human infection model (CHIM; aka, human challenge studies)^12^ can also be used as the study population. As compared to a naturally infected population, a CHIM has advantages and disadvantages. The advantages include the ability to control the population being infected regarding their naturally acquired immunity, co-infections, genetics, microbiome, nutrition, and environment, as well as the ability to control the timing, route, and/or dose of the infection and the infecting microorganism so that no disease is caused or the disease is self-limiting or can be controlled with cures or treatments^13^. The knowledge of the time of the infection allows a detailed characterization of the post-infection time course of the host immune response. The ability to control the pathogen dosage enables the study of pathogen dosage effects on the clinical and immune responses^14^. Importantly, the ability to collect pre-exposure samples provides a background to understand the correlate of protection, and whether it may differ between symptomatic primary infection and symptomatic re-infection. Because of these benefits, there has been an increase in calls for CHIMs in areas where study volunteers may have had prior exposure to the pathogen being studied and other pathogens^15–17^. Additionally, as compared to trials of vaccines in high attack-rate populations, CHIMs can be smaller, shorter, less expensive, and expose fewer participants to experimental vaccines^13^. Furthermore, CHIMs can be valuable for identifying lead vaccine candidates to test in larger studies, and thereby accelerate vaccine development to realize public health benefit sooner, strengthening the ethical rationale against the risk of harming participants^18^.

However, CHIMs have disadvantages, as previously described by Abo et al. ^12^ and summarized here: (1) The challenge strain may poorly represent naturally circulating pathogen strains, so the efficacy and correlate of protection of the vaccine may not extrapolate to field settings; (2) For safety reasons, challenge models use disease of low severity that might not apply to disease of greater severity in natural populations; (3) A high pathogen inoculum to achieve a high attack rate can overwhelm a vaccine’s protection, and low attack rates in placebo arms have affected efficacy rate determinations; and, (4) As compared to the target population, CHIM participants can differ in comorbidities, immunity, age, immunogenetics, and microbiome, which can influence a vaccine’s efficacy and correlate of protection. For a correlate of protection identified in a CHIM to be used to guide vaccine development, it needs to be validated in a field trial, typically a phase 2b study^19^. CHIMs that overcome these potential difficulties with participants, pathogens, and diseases, as with studies of natural high attack-rate subpopulations, offer the potential to de-risk and accelerate late-stage vaccine development.

## Bridging from the high attack-rate population to other populations

**Fig. 4:**
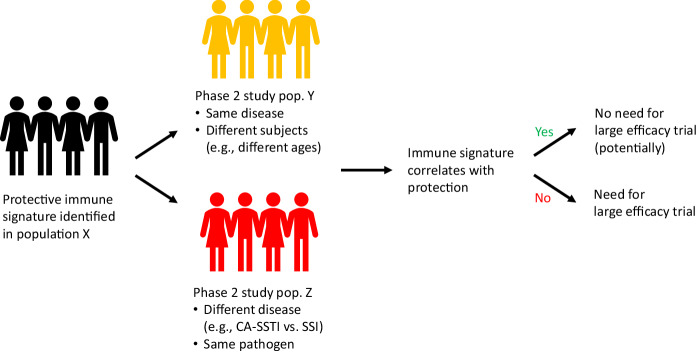
Extending proof-of-principle efficacy to other populations.

A vaccine that prevents a disease in one population generally requires a phase-3 efficacy trial to demonstrate efficacy in another population. Such a requirement for multiple phase-3 trials burdens clinical development programs to the point of infeasibility in some cases, especially when a low incidence of infection demands trials of thousands of subjects. However, a Reverse Vaccine Development study design can potentially identify a correlate of protection that can be used as an immunological bridge to assess a vaccine’s potential efficacy in other populations. When populations differ mainly in the characteristics of patients (e.g., age, health status, ethnicity, gender) or the cause of the infection (e.g., community acquired pneumonia vs. ventilator-associated pneumonia), but the disease manifestation is largely the same, the validation of the correlate of protection in subsequent populations may eliminate the need for large efficacy trials (Fig. 4).

However, some pathogens can cause quite different diseases. For example, *S. aureus* can cause CA-SSTI, Surgical Site Infection (SSI), Bloodstream Infection (BSI), and pneumonia — and a vaccine’s efficacy against these diseases may vary. Similarly, *P. aeruginosa*, *E. coli*, *K. pneumoniae, Enterococcus spp*., *S. pyogenes*, *A. baumannii*, and *Candida spp*. can each cause different diseases.

In such instances, a Reverse Vaccine Development study design that identifies a correlate of protection can still provide value. For example, the correlate of protection could enable a reduced sample size. The correlate can be assessed in a small number of patients with the different disease manifestation. If the immune correlate is seen in the infected patients, the vaccine may not protect against the different manifestation, and the efficacy assessment may require a larger enrollment. Alternatively, if the infected participants show no correlate, but most vaccinees show the correlate and remain uninfected, then the infected participants likely had a suboptimal immune response, and dose escalation may be needed.

The development of a vaccine without a correlate of protection leaves only arbitrary immunological endpoints on which to base the selection of vaccine dosage, schedule, and adjuvant. Conversely, a known immune correlate can be used early in clinical development to optimize these elements that may be crucial for improving vaccine efficacy.

## Limitations

Reverse Vaccine Development requires the availability of a suitable surrogate population that may be a naturally occurring, high attack-rate population or a human challenge study, which is possible for some pathogens.

An early efficacy indication in a surrogate population reduces risk for phase-3 study failure. However, the identification of a correlate of protection remains challenging despite the increasing numbers of immune parameters and machine learning algorithms to assess signatures of protection. Should an immune signature be identified by cross-validation in a relatively small, high attack-rate population with a defined immune background, and it is validated in a holdout or matched test population, its relevance to the population intended for marketing approval remains a risk.

A naturally infected population used for Reverse Vaccine Development comprises hundreds of study participants with each participant providing several longitudinal samples for assessing multiple immune parameters, which adds cost exceeding that of a conventional phase 1/2 study. However, we estimate that the risks and cost are worth the potential benefits of having a proof-of-concept correlate of protection to guide the selection of vaccine dosage, schedule, and adjuvant, and to provide an immunological bridge to assess vaccine efficacy in other populations, such as those where a large phase 3 clinical trial may not be feasible.

## Conclusion

Reverse Vaccine Development assesses vaccine efficacy in the early phases of clinical development in a high attack-rate population and has the potential to identify a correlate of protection. Alternatively, depending on the pathogen, a CHIM may serve as the high attack-rate population. Early identification of proof-of-concept efficacy lowers the risk of failure in large phase-3 trials. Additionally, a proof-of-concept efficacy demonstration offers the opportunity to explore immune correlates of protection in a relatively small-sized trial, using machine learning to integrate multiple immune readouts. If an immune correlate is confirmed, it can guide the vaccine dosage, schedule, and adjuvant to improve the immune response and efficacy. Additionally, an immune correlate identified early in clinical development in a high attack-rate population can be used to corroborate efficacy in other populations that differ in characteristics such as disease incidence, age, health status, ethnicity, or disease manifestation (e.g., CA-SSTI or SSI). Reverse Vaccine Development has the potential to facilitate the development of vaccines against AMR pathogens.
